# Chimeric antigen receptor T-cell therapy for T-ALL and AML

**DOI:** 10.3389/fonc.2022.967754

**Published:** 2022-11-29

**Authors:** Wenwen Wei, Dong Yang, Xi Chen, Dandan Liang, Liqun Zou, Xudong Zhao

**Affiliations:** ^1^ Laboratory of Animal Tumor Models, Frontiers Science Center for Disease-Related Molecular Network, State Key Laboratory of Biotherapy and Cancer Center, National Clinical Research Center for Geriatrics, West China Hospital of Sichuan University, Chengdu, China; ^2^ Department of Medical Oncology of Cancer Center, West China Hospital of Sichuan University, Chengdu, China; ^3^ Department of Radiotherapy, Cancer Center, West China Hospital of Sichuan University, Chengdu, China

**Keywords:** chimeric antigen receptor, T-ALL, AML, antigen, immunotherapy

## Abstract

Non-B-cell acute leukemia is a term that encompasses T-cell acute lymphoblastic leukemia (T-ALL) and acute myeloid leukemia (AML). Currently, the therapeutic effectiveness of existing treatments for refractory or relapsed (R/R) non-B-cell acute leukemia is limited. In such situations, chimeric antigen receptor (CAR)-T cell therapy may be a promising approach to treat non-B-cell acute leukemia, given its promising results in B-cell acute lymphoblastic leukemia (B-ALL). Nevertheless, fratricide, malignant contamination, T cell aplasia for T-ALL, and specific antigen selection and complex microenvironment for AML remain significant challenges in the implementation of CAR-T therapy for T-ALL and AML patients in the clinic. Therefore, designs of CAR-T cells targeting CD5 and CD7 for T-ALL and CD123, CD33, and CLL1 for AML show promising efficacy and safety profiles in clinical trials. In this review, we summarize the characteristics of non-B-cell acute leukemia, the development of CARs, the CAR targets, and their efficacy for treating non-B-cell acute leukemia.

## Introduction

### Clinical features, treatment, and prognosis of T-ALL

T-ALL is a highly invasive form of hematological malignancy that results from the malignant transformation of immature T-cell progenitors, characterized by active cell proliferation, high tumor burden, leucocyte count, extramedullary involvement, large thymic masses, and pleural effusions. T-ALL occurs in 10%-15% of pediatric and about 25% of adult ALL cases, respectively (1). Compared with B-ALL, T-ALL cases are generally diagnosed in older individuals, are biologically distinct to B-ALL and have different kinetic patterns of disease response. For example, most B-ALL originates from the pre-pro-B and pro-B-cell, while, in contrast, T-ALL originates from various stages of T cells. In addition, T-ALL patients are generally more resistant to conventional chemotherapeutic drugs than patients with B-ALL. Notably, B-ALL has also been found to be associated with favorable (low-risk) genetic subtypes that inform reliable therapeutic implications and realistic prognostication of their condition in patients, thereby facilitating risk stratification and targeted therapy (2–6). However, given the greater genetic and cellular heterogeneity, such an approach is so far elusive for T-ALL, with current treatment approaches relying on multidrug combination followed by intensive consolidation and maintenance therapy, with central nervous system (CNS) prophylaxis given at intervals throughout treatment (7). Nevertheless, treatments for T-ALL show significant success, with 5-year survival rates of 80%-90% for pediatric cases, yet 30%-40% for adult cases (8, 9), but there are still 20% of T-ALL patients ultimately die because of relapsed or refractory disease. The development of radiotherapy, new drugs, and targeted therapies targeting CD19, CD20, and CD22, have altogether improved the clinical management of R/R B-ALL. In contrast, curative treatments for R/R T-ALL remain to be significantly found, with hematopoietic stem cell transplantation (HSCT) currently the only such approach.

### Clinical features, treatment, and prognosis of AML

AML is a hematological malignancy formed by abnormal clonal proliferation of primitive myeloid cells, and characterized by the accumulation of deformed, immature, and nonfunctional myeloid cells in bone marrow and blood. The incidence of AML increases with age, accounting for 15%-20% of leukemia cases in childhood, and is the second most common form of leukemia in children while it is the most common adult acute leukemia (10). In most children, AML often occurs *de novo* while in adult, a major proportion of AML are generally preceded by myeloproliferative neoplasms (MPN) or myelodysplastic syndrome (MDS) (11, 12). Current strategies for the treatment of AML involve two-phase chemotherapy, including anthracycline- and cytarabine-based induction chemotherapy, wherein children also receive central nervous system (CNS) prophylaxis to prevent central nervous system relapse. Patients who achieve initial remission then receive consolidation/intensification therapy, including combination chemotherapy or HSCT. While the treatment for children with acute promyelocytic leukemia (APL) includes a third phase called maintenance, which gives lower dose treatment than those used during the induction and consolidation phases (13–15). After treatment with standard regimens, the long-term survival rate of AML approaches near 70% in children and 35-45% in adult patients under 60 years, compared with 10-15% for those over 60 years (16). For relapsed patients, the median survival is 6 months and approximately 10% of patients achieve long-term survival, relapse and associated complications are common causes of death in AML (17, 18). Most recently, targeted therapy, immunotherapy, and new drugs have provided more treatment options for AML, but the efficacy in R/R patients remains poor. It is an urgent need for more effective treatments to improve patient survival rates for AML.

## The structure of CARs

**Figure 1:**
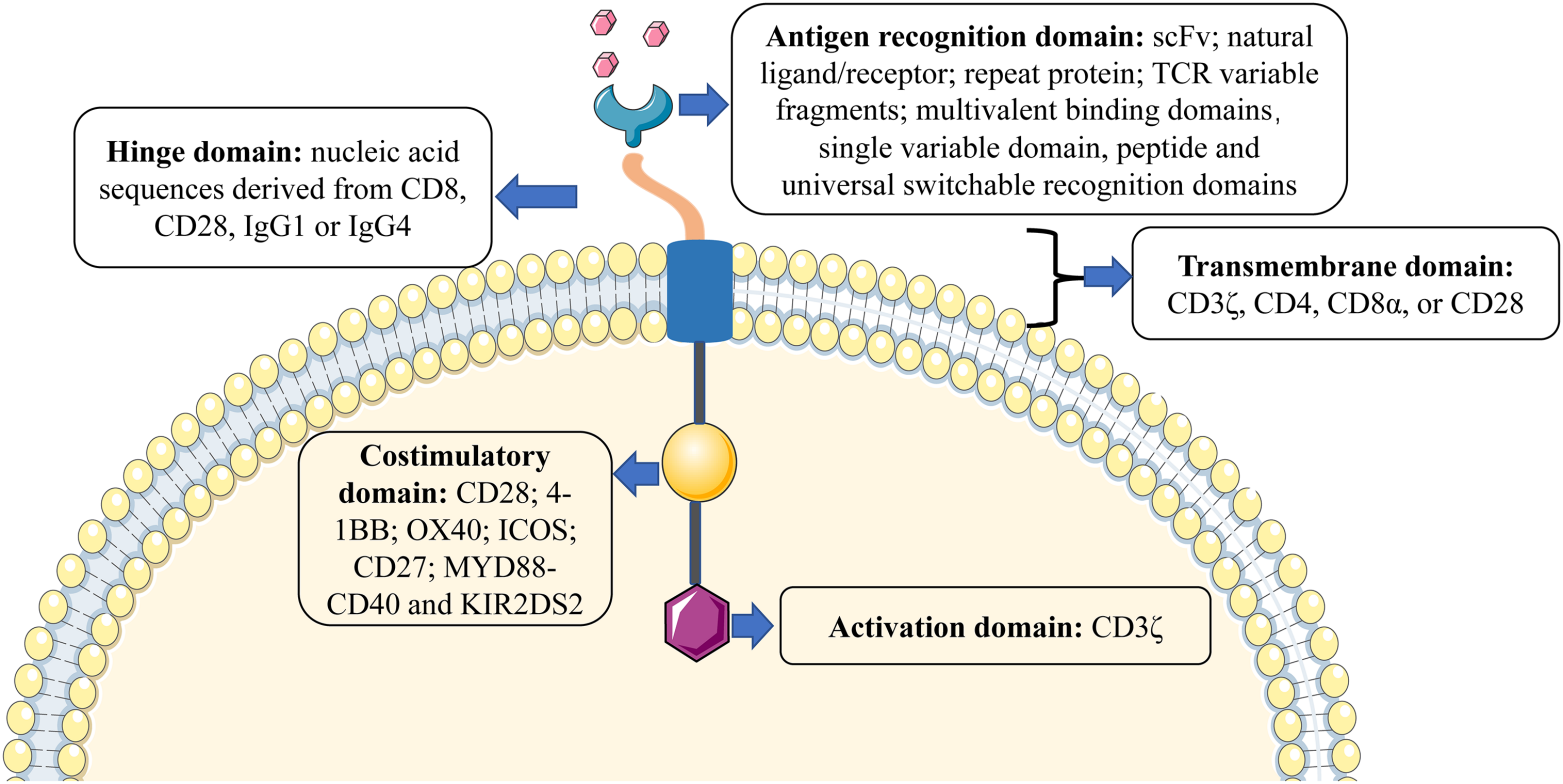
The basic structure of CARs.

CARs are various receptors that endow T cells with the capacity to recognize specific tumor antigens and induce cytotoxicity against malignant cells, based on their expression of such antigens (19). The basic structure of CARs consists of four components (as shown in **Figure 1**): (1) Antigen recognition domain. The antigen recognition domain is the extracellular domain of the CAR, which is essential for T-cell activation, recognition, and cytotoxicity. The most common extracellular antigen recognition domain is a single-chain variable fragment (scFv) composed of heavy (V_H_) and light (V_L_) chains derived from monoclonal antibodies. In addition, natural ligands or receptors, repeat proteins such as designed ankyrin-repeat proteins (DARPins) and variable lymphocyte receptors (VLRs) derived from the sea lamprey genome, T-cell receptor (TCR) variable fragments, multivalent binding domains, universal switchable recognition domains, single variable domain on a heavy chain such as nanobody (also referred as VHH) and peptide, as well as others, could be used to construct the CAR (20, 21). (2) Hinge domain. The hinge domain is an extracellular structure between the antigen recognition domain and the transmembrane domain. The length and composition of the hinge domain are known to affect the flexibility of the CAR, CAR expression, signal transduction, and epitope recognition (22, 23). Presently, the most commonly effective hinge domains for CAR design comprise amino acid sequences derived from CD8, CD28, IgG1, or IgG4 (24). (3) Transmembrane domain. The transmembrane domain anchors the CAR to the cell surface membrane and is frequently derived from type I proteins including CD3ζ, CD4, CD8α, and CD28. Different transmembrane domains influence the stability and function of CARs. For example, CD3ζ mediates CAR dimerization, and the insertion of endogenous TCRs can promote CAR-mediated T-cell activation (25), but it is also less stable compared to the CD28 transmembrane domain (26). The transmembrane domain derived from CD8α induces less IFNγ and TNFα release than CD28 and is less sensitive to activation-induced cell death (AICD) (27). (4) Intracellular signal domain. This region is composed of a typical intracellular signal domain that includes an activation domain, as well as one or more costimulatory domains. Current CARs activate T-cells through the CD3ζ-derived immunoreceptor tyrosine-activated domain (24). However, the activation domain alone may not be enough to induce an effective response of CAR-T cells, since the persistence and activity of CAR-T cells *in vivo* remain limited (28). Costimulatory domains combined with the activation domain-bearing CD19-targeting CAR-T cells result in better persistence in B-cell malignancies (29). The two most common costimulatory domains, CD28 and 4-1BB (also known as CD137 or TNFRSF9) are used in most clinical trials and CD28-bearing and 4-1BB-bearing CD19 CAR have been approved by Food and Drug Administration (FDA) for B-cell malignancies and achieved promising clinical responses (30–35). Further, other costimulatory domains, such as OX40 (also known as CD134), ICOS (inducible T-cell costimulator), CD27, MYD88-CD40, and KIR2DS2 (killer cell immunoglobulin-like receptor 2DS2), that have demonstrated efficacy in preclinical models but have not yet been validated in clinical studies (36–40).

## The evolution of CAR development

**Figure 2:**
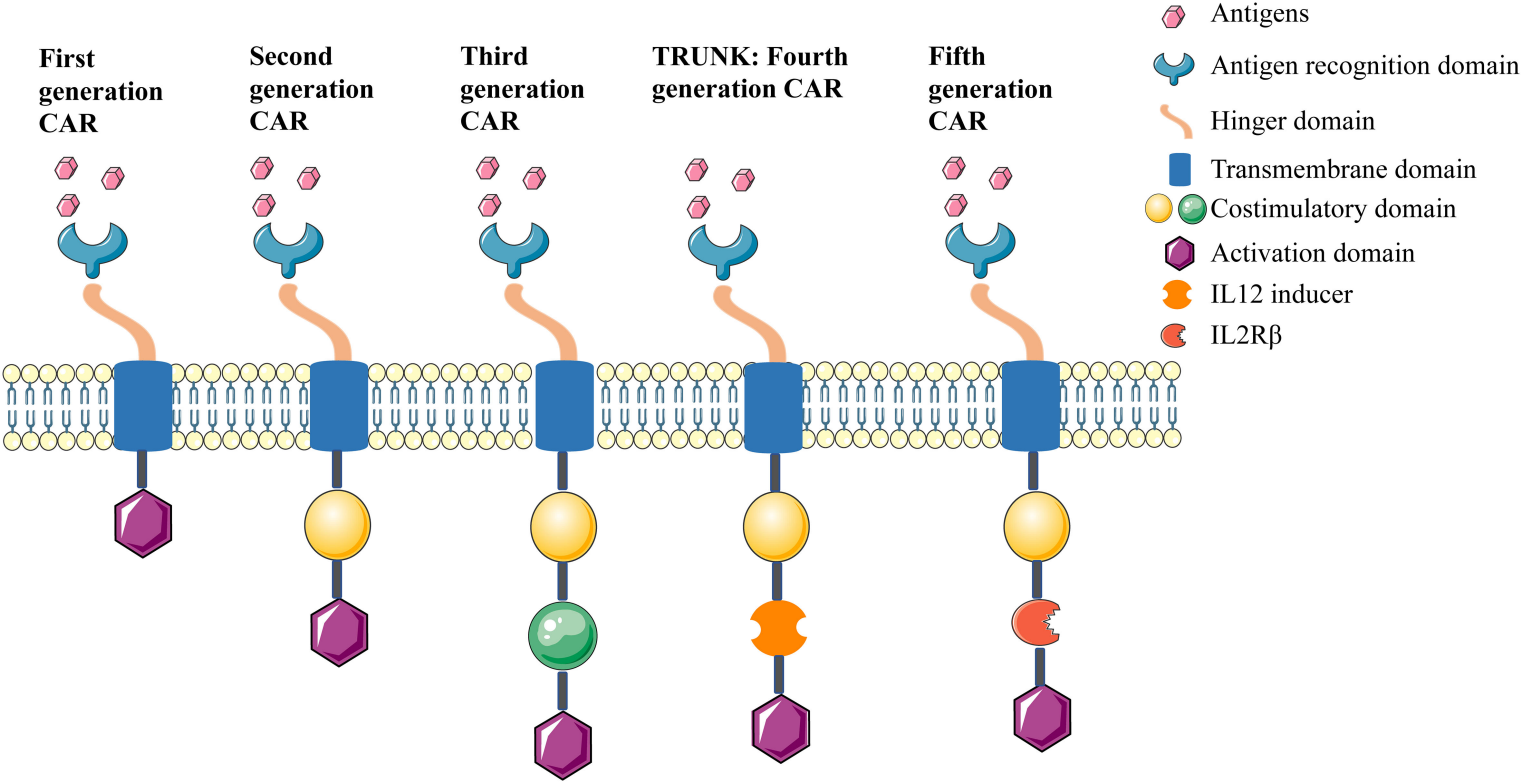
Five generation of CARs.

Since the first generation CAR was described in the late 1990s; four further generations of CARs have been developed, (summarised in **Figure 2**). The first generation CAR contained the CD3ζ intracellular signaling domain but without costimulatory domains, and it induced low interleukin (IL)-2 production and displayed inadequate proliferation and short lifespan *in vivo* (41). From this, the second generation CAR comprised an additional costimulatory domain, such as CD28, 4-1BB, or OX-40, and this led to enhanced proliferation, cytotoxicity, and persistence for CAR (42). The third generation CAR combined multiple costimulatory signaling domains. Although these represent a good safety profile in tumor therapy, their efficacy was not significantly improved compared with the second generation CAR (43). The fourth generation CAR refers to T-cells redirected for universal cytokine-mediated killing (TRUCKs), which added IL-12 based on the second generation CAR. In this design, IL-12 is expressed either constitutively or inducibly after CAR activation, which promotes the production and secretion of desired cytokines, as well as enhances cytotoxicity against tumor cells through multiple synergistic mechanisms (44, 45). The fifth generation CAR design involved the addition of a β-chain domain of the IL-2 receptor based on the second generation CAR, which comprises a binding site for the transcription factor STAT3. Antigen-specific activation of this receptor can trigger three signals: the TCR *via* CD3ζ domains, the costimulatory domain *via* the CD28 domain, and cytokine signaling *via* JAK-STAT, which act synergistically to activate and expand CAR-T cells (46, 47).

## The advantage of CAR-T therapy

When compared with TCRs, CARs are major histocompatibility complex (MHC) independent and can recognize targeted antigens expressed on the cell surface (48, 49), which is in contrast to TCRs that only recognize natural antigens presented by MHC (50). Thus, loss of MHC class I is recognized as the major mechanism of immune escape for tumor cells. As such, the characteristic of MHC independence makes CAR-T cells more applicable for tumor therapy (51). CAR-T cells eliminate tumor cells by recognizing tumor-specific antigens (TSA) on the surface of tumor cells, which has the advantage of minimizing damage to normal tissues (52, 53). In addition, tumor cells downregulate the expression of costimulatory molecules, and the intracellular structure of CAR contains a costimulatory domain that counteracts this effect, leading to improved therapeutic efficacy for treating tumors. It is noteworthy that CAR not only recognizes protein antigens but also recognizes carbohydrates and lipids antigens, thereby providing more design options for the preparation of effective CAR (54, 55).

## CAR-T therapy for non-B-cell acute leukemia

**Figure 3:**
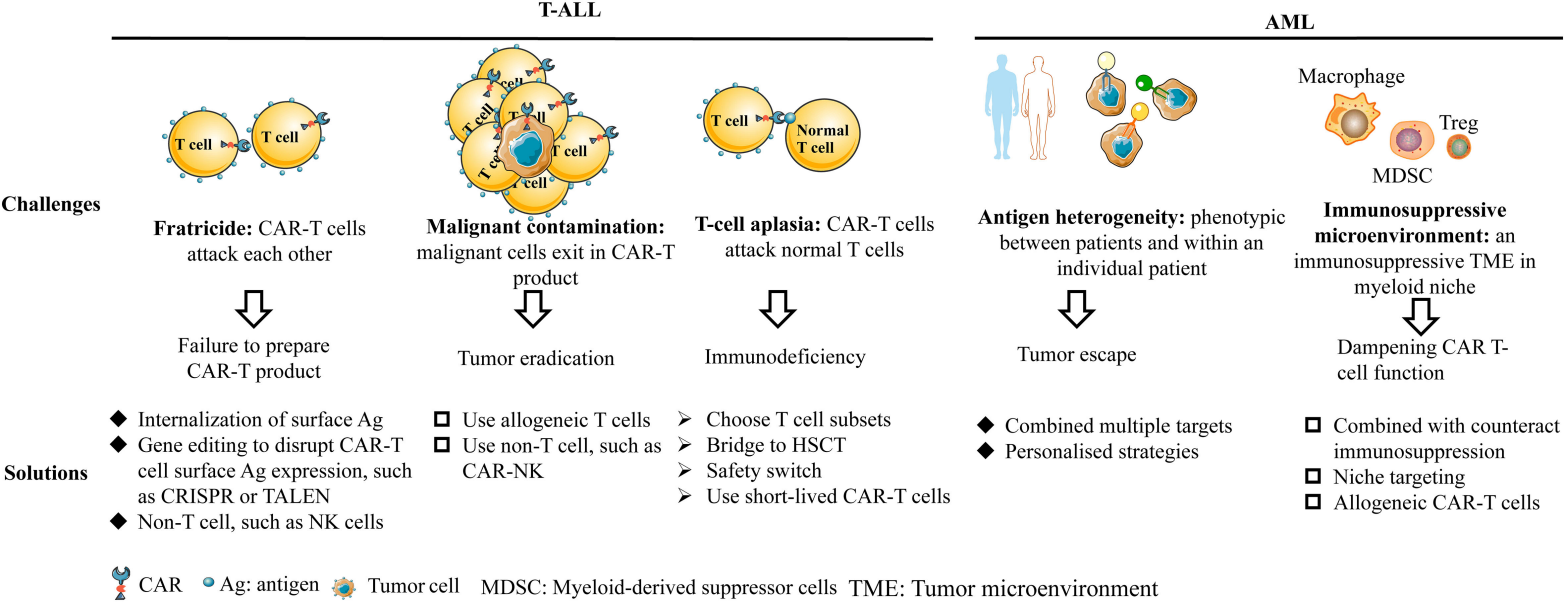
The challenges and solutions for T-ALL and AML.

The development of CAR-T therapy for non-B-cell acute leukemia faces some unique challenges, such as fratricide, malignant contamination, T-cell aplasia for T-ALL and antigen heterogeneity, and immunosuppressive environment for AML. Although there are obstacles in the development of CAR-T therapy for non-B-cell acute leukemia, some strategies have been developed to solve these problems, as shown in **Figure 3**. In this review, we focus on specific targets with promising efficacy and safety, which have been verified in preclinical or clinical trials.

### Antigen targets of CAR-T therapy in T-ALL

#### CD5

CD5 is a surface marker of T-cell malignancies and is expressed in approximately 80%-95% of T-ALL or T lymphoblastic lymphoma (TLL) (56, 57). Typically, CD5 is also expressed on mature peripheral blood T cells, thymocytes, and some B-cell lymphocytes in healthy tissues, which lead to fratricide of CAR-T cells (58, 59). In 2015, Mamonkin et al. reported that CD5 CAR-T cells only exhibit partial fratricide, following which these could be expanded *in vitro*. The expanded CAR-T cells also maintained killing efficacy in T-ALL/TLL tumor cell lines (including Jurkat, CCRF-CEM, MOLT4, Hut78, and SupT1) and primary T-ALL cells *in vitro* and Jurkat and CCRF-CEM cell lines *in vivo*. Further investigation found that the incomplete fratricide of CD5 CAR-T cells was due to the internalization of surface CD5 molecules after ligand binding which, in turn, downregulated CD5 expression on the normal T-cell surface (60). To prevent fratricide completely, in 2017, Raikar et al. genetically knocked out CD5 expression on the surface of T cells with CRISPR-Cas9 genome editing, and CD5-edited effector T cells overcame the challenge of self-activation and fratricide, which demonstrating the feasibility for CD5 CAR-T therapy in T-cell malignancies (61). To prolong the persistence of CAR-T cells *in vivo*, in 2018, Mamonkin et al. designed a doxycycline (*Dox*) controlled Tet-Off system to inhibit CAR expression to prevent fratricide of 4-1BB CD5 CAR instead of CD28 CD5 CAR. In this study, CD5 expression in CAR-T cells occurred after *Dox* withdrawal, leading to improved and prolonged antitumor ability against CD5+ T-ALL cell lines Jurkat and CCRF-CEM *in vitro*, as well as the Jurkat mouse model *in vivo (* 62). To explore the feasibility of CD5 CAR-T therapy as a bridge to HSCT in the clinic, in 2019, Hill et al. treated 9 heavily treated patients with autologous CD5 CAR-T cells, and 3/9 patients achieved CR, 4/9 patients obtained an objective response, and 2/9 patients relapsed at 6 weeks and 7 months post-infusion, all side-effects were manageable. These results proved that CD5 CAR-T cells could allow ineligible patients to proceed to HSCT with safety and clinical response for R/R CD5+ malignancies (63). Except for the fratricide, T-cell aplasia is also an important challenge for CD5 CAR. In 2020, Wada et al. used alemtuzumab, which targets CD52 as an inducible safety switch, to remove CAR-T cells from systemic blood circulation without affecting the anti-tumor efficacy in the mouse model, thus avoiding T-cell aplasia after therapy (64). T-cell malignancies with CNS infiltration always have poor outcomes and limited treatment options, IL-15 could strengthen the anti-tumor response. In 2021, Feng et al. modified a CD5-IL-15/IL15sushi CAR which secretes an IL-15/IL-15 complex, to explore its clinical effectiveness against one refractory T-cell lymphoma patient with CNS infiltration. This patient obtained a rapid ablation of the CNS lymphoblast and lymphoma and was accompanied by brief and transient T-cell aplasia (65). In another study, Dai et al. manufactured a new biepitopic CAR with fully human heavy-chain variables FHV_H_3 and FHV_H_1, which could bind different epitopes of CD5. As such, CD5KO FHV_H_3/V_H_1 CAR-T cells showed prolonged and sustained efficacy against CD5+ T-ALL cell lines, such as Jurkat, CCRF-CEM, MOLT4, SupT1 *in vitro*, and CCRF-CEM *in vivo*, with moderate cytokine production, proved that this new CD5 CAR deserved more exploration (66). Due to recurrence occurring in some patients after CAR-T therapy which targets single antigen CD5 or CD7 (63, 67), in 2022, Dai et al. designed a bispecific CAR that targeted CD5 and CD7 with a fully human variable heavy chain (FHV_H_). The results showed that fratricide-resistant FHV_H_-derived CD5/CD7 bispecific CAR-T cells showed potent antitumor activity to Jurkat, CCRF-CEM, MOLT-4, and SUP-T1 cell lines *in vitro* and CCRF-CEM T-ALL mouse model *in vivo*, it provided the possibility to the populations with antigen heterogeneous (68). Pan et al. explored the safety and efficacy of donor-derived CD5 CAR-T cells in CD7-negative relapsed patients after CD7 CAR therapy. The data showed that all five patients achieved CR at 1 month and no dose-limiting toxicities occurred. Donor-derived CD5 CAR showed promising clinical safety and response in R/R T-ALL, but the evaluation of durable remission and functional immune system reconstitution needs longer follow-ups (69). Possible approaches to overcome the challenges such as fratricide and T-cell aplasia include internalization of surface CD5, CRISPR-Cas9, or Tet-Off system technologies that could be used to deal with complete fratricide without affecting the efficacy of CAR-T cells, while alemtuzumab could be used as a safety switch to deplete CAR-T cells after therapy to prevent T-cell aplasia. Taken together, CD5 is a promising target for CD5+ hematological malignancies, there are 4 clinical trials about CD5 CARs that are being recruited, as shown in **Table 1**.

**Table 1:** Recruiting for CD5 CAR clinical trials.

NCT Number	Interventions	First posted	Phase	Locations
NCT03081910	Autologous CD5.CAR/28 zeta CAR T	16-Mar-17	I	United States
NCT04594135	anti-CD5 CAR T	20-Oct-20	I	China
NCT05032599	Donor-derived CD5 CAR T	2-Sep-21	I	China
NCT05487495	Donor-derived CD5 CAR T (CT125B)	4-Aug-22	I	China

#### CD3

CD3 is a pan-T-cell surface antigen, expressed predominantly on all mature T-cells (70), however, the development of CAR-T targeting CD3 remains limited due to the complete fratricide of CAR-T cells. In 2018, Rasaiyaah et al. tried to disrupt endogenous TCRαβ/CD3 on T cells by adopting transcription activator-like effector nuclease (TALEN) to construct a CAR targeting CD3ϵ and CAR-T cells exhibited specific toxicity against the T-ALL cell line Jurkat *in vitro* and *in vivo (* 71). However, since T-ALL and TLL cells derived from patients typically express cytoplasmic CD3 (cCD3) rather than membrane CD3 (mCD3) (72), the therapeutic effect of CAR targeting CD3 is limited in its clinical application. No clinical trials are currently documented in the literature.

#### CD7

CD7 is a 40-kD Ig superfamily member expressed on normal T and NK cells (73–75) and is expressed in over 95% of ALL and 30% of AML, as well as some lymphomas (56, 76–79). CAR-T cells targeting CD7 showed complete fratricide and could not be expanded, thereby resulting in limited studies of CAR targeting CD7 until 2017. Gomes-Silva et al. first knocked out CD7 expression on normal T cells by CRISPR–Cas9 pre-T-transfection so that CD7-knocked out CAR-T cells could be expanded, and these cells showed specific cytotoxicity to both CD7+ cell lines Jurkat, CCRF-CEM, MOLT-4, Hut78 and SupT1, also primary blasts, these provided a new insight for CD7 CAR (76). Png et al. reported a study that blocked CD7 in the ER/Golgi by using a protein expression blocker (PEBL) system, in which CD7 could not be expressed on the surface of T cells. This method alleviated fratricide without affecting the proliferation of CAR-T cells, while CD7 CAR-T cells showed strong antileukemia activity to CD7+ cell lines including Jurkat, CCRF-CEM, Loucy, MOLT4, and KG1a (80). Due to CD7 expression on both normal and malignant T cells, fratricide and malignant contamination occurred during CAR-T cell preparation. To solve both issues, allogeneic T cells may be another choice to prepare CAR-T cells. Then in 2018, Cooper et al. knocked out CD7 and the T-cell receptor alpha chain (TRAC) of T cells by CRISPR–Cas9, and CD7 CAR-T cells showed antileukemia efficacy against T-ALL cell lines MOLT-3, MOLT-4, HSB-2, and CCRF-CEM without graft-versus-host-disease (GVHD) (81). In 2020, Zhang et al. explored the antitumor activity and toxicity of CD7 CAR in clinical trials for the first time. The author reported that 2 of 3 enrolled R/R ALL/TLL patients obtained complete remission (CR) with minimal residual disease (MRD) negative at day 28 post-infusion. At the same time, cytokine release syndrome (CRS) occurred which was controllable. Nevertheless, this study showed the significant potential value for the clinical application of CD7 CAR (82). To address CRS after CAR-T cell therapy, in 2021, Li et al. treated 2 T-ALL patients with “off-the-shelf” allogeneic CD7 CAR-T cells combined with ruxolitinib. Strong CAR-T-cell expansion and rapid tumor cell clearance were detected after CAR-T cell infusion, and both patients achieved CR with MRD negative. While, both patients developed grade 3 CRS, but it was manageable with co-treatment of ruxolitinib (83). Yang et al. conducted a phase I clinical trial of CD7 CAR-T cell therapy for 14 R/R T-ALL. The data showed that 13/14 (92.9%) patients achieved MRD negative CR at day 28 post-infusion and all patients experienced mild CRS (grade<2) (84). For T-ALL patients, there may be not enough normal T cells for CAR-T cells preparation, Pan et al. used donor-derived CD7 CAR-T cells to explore the efficacy and safety in R/R T-ALL, 18/20 patients achieved CR with 7 patients proceeding to HSCT and with a manageable safety profile (67). Furthermore, CAR-T/NK targeting CD7 will clear CD7-positive normal T/NK cells *in vivo*, HSCT is usually required after CAR-T therapy; otherwise, the treatment causes severe immunodeficiency and potentially life-threatening infection by pathogenic microorganisms. Kim et al. knocked out CD7 in hematopoietic stem cells (HSCs) and found that these CD7-KO HSCs differentiated into CD7-negative T-cells and NK cells that exhibited effector functions after transplantation into mice. This study suggests that such an approach could resolve T-cell immunodeficiency caused by CD7-CAR therapy, altogether providing a potential new approach for the development of CD7 CAR (85). In 2022, Dai et al. reported a case that haploidentical CD7 CAR-T cells induced remission in an 11-year-old TP53 mutated R/R ETP-ALL/LBL patient, and grade 3 CRS and macrophage activation syndrome were observed but manageable (86). Due to the GVHD occurring in some patients after donor-derived CD7 CAR-T cell therapy, Zhao et al. treated five R/R T-ALL/LBL with autologous CAR-T cell therapy. The results showed that 4/5 patients achieved CR at day 30 post-infusion without neurotoxicity, GVHD, or infection (87). Due to 3-10% CD7- T cells exiting in peripheral blood, Lu et al. obtained naturally selected 7CAR (NS7CAR) T cells, which are a subtype of CD7 negative T-cells that survived from fratricide. The NS7CAR T cells comprised a higher proportion of CAR+ cells and CD8+ central memory T cells while maintaining similar therapeutic activity to cell line CCRF-CEM *in vitro* and *in vivo* when compared with CD7 knocked-out 7CAR T cells. Most importantly, a study of 14 R/R T-ALL and 6 T-LBL patients who received NS7CAR T therapy found that 19/20 patients achieved MRD CR in bone marrow at day 28, all with manageable side effects (88). In another study, Freiwan et al. sorted CD7- T cells from PBMC before transducing them with CD7 CAR, the CD7- CAR-T cells contained more CD4+ memory phenotype and have a robust antitumor activity to CD7+ cell lines CCRF-CEM and MOLT3 *in vitro* and eliminated CCRF-CEM cells in the mouse model, as well as bypass fratricide (89). Li et al. combined donor-derived CD7 CAR-T therapy with allogeneic HSCT for a 3-year-old hepatitis B-positive T-ALL patient. The patient had CR at seven months post-infusion and the copy number of hepatitis B virus continuously decreased during treatment (90). Hu et al. resisted fratricide, GVHD, and allogeneic rejection in healthy donor-derived CD7 CAR (RD13-01) by genetic modifications. Twelve patients were recruited in the phase I clinical trial, and the data showed that 81.8% of patients achieved objective responses and 63.6% of patients received CR with no dose-limiting toxicity, GVHD, neurotoxicity, or severe CRS (91). CD7 CAR showed satisfied efficacy and safety in various clinical trials, gene editing technologies such as CRISPR-Cas9, TALEN, or PEBL, and recently reported natural selection could be applied to overcome fratricide, while HSCT could be applied in combination with CAR to address potential T-cell aplasia, and CRS could be controlled by ruxolitinib. Taken together, CD7 CAR is a promising therapeutic target for R/R T-cell malignancies. Currently, there are 23 clinical trials of CD7 CAR recruiting to explore its therapeutic effects in T-cell malignancies, as shown in **Table 2**.

**Table 2:** Recruiting CD7 CAR clinical trials.

NCT Number	Interventions	First posted	Phase	Location
NCT04033302	CD7 CAR gene-engineered T	26-Jul-19	I/II	China
NCT05127135	Allogeneic CART7	19-Nov-21	I	China
NCT04702841	CD7 CAR-γδ T	11-Jan-21	I	China
NCT04620655	RD13-01 cells	9-Nov-20	NA	China
NCT04480788	CD7-CART	21-Jul-20	I	China
NCT04916860	Senl-T7	8-Jan-21	NA	China
NCT04689659	Donor-derived CD7 CAR-T	30-Dec-20	II	China
NCT04264078	CD7 UCAR-T	11-Feb-20	I	China
NCT04785833	autologous CD7 CAR-T	8-Mar-21	NA	China
NCT04823091	CAR7-T Cells	30-Mar-21	I	China
NCT04840875	Autologous CD7 CAR-T	12-Apr-21	I	China
NCT04938115	CD7 CAR-T	24-Jun-21	NA	China
NCT04762485	Humanized CD7 CAR-T	21-Feb-21	I/II	China
NCT03690011	Autologous CD7.CAR/28zeta CAR-T	1-Oct-18	I	United States
NCT05043571	anti-CD7 CAR-T	14-Sep-21	I	Singapore
NCT05170568	PA3-17 CAR-T	28-Dec-21	I	China
NCT04984356	WU- CART-007	30-Jan-21	I/II	United States
NCT04934774	Non-gene edited CD7 CAR-T	22-Jan-21	I	China
NCT04004637	CD7 CAR-T	2-Jul-19	I	China
NCT04599556	anti-CD7 CAR-T	22-Oct-22	I/II	China
NCT05212584	CD7CAR-T	28-Jan-22	I	China
NCT05290155	Anti-CD7 CAR-T	22-Mar-22	I	China
NCT05398614	Senl 101	1-Jun-22	I	China

“NA” means “Not Applicable”.

In addition to CARs targeting T-cell pan-antigens, some antigens are only expressed on a subset of T cells, which can avoid complete fratricide during CAR-T preparation and T-cell immunodeficiency during therapy.

#### CD4

CD4 is expressed in most TLLs and some T-ALLs and its expression is restricted to the hematopoietic compartment but it is not expressed in HSCs. As the target of CAR, it can potentially reduce the off-target side effects for non-hematological tissues. But the persistence of CD4 CAR T cells after removal of tumor cells can lead to aplasia of CD4 positive T cells and cause HIV/AIDS-like syndrome. In 2016, Ma et al. utilized CAMPATH (alemtuzumab) as a natural safety switch to deplete CD4 CAR T cells post-therapy in mice. They found that this approach was effective to kill Jurkat cells *in vitro* and *in vivo* with minimal toxic side effects from loss of CD4+ T-cells (92). CD4 is expressed on a subset of normal T cells, which could prevent complete fratricide and be used to kill CD4 high-expressed malignant T cells. Three CD4 CARs are currently being recruited to explore clinical efficacy in T-ALL, as shown in **Table 3**.

**Table 3:** Recruiting CD4 CAR clinical trials.

NCT Number	Interventions	First posted	Phase	Locations
NCT04162340	CD4 CAR T	14-Nov-19	I	China
NCT04219319	LCAR-T2C CAR T	7-Jan-20	I	China
NCT04973527	LCAR-T2C CAR T	22-Jul-21	I	China

#### CD1a

The expression of CD1a is largely restricted to developing cortical thymocytes, and neither CD34+ progenitor cells nor T-cells are expressed during ontogeny. In contrast, however, CD1a is only expressed in cortical T-ALL (coT-ALL) and its expression persists in relapsed patients. Diego et al. found that CD1a CAR-T cells are fratricide-resistant and showed good efficacy against CD1a+ Jurkat and MOLT4 cell lines and primary coT-ALL cells *in vitro* and Jurkat T-ALL mouse models *in vivo (* 93). Although CD1a CAR could be used to treat coT-ALL, some limitations exist to CD1a CAR in clinical use, such as that only a minority of cases of T-ALL express CD1a. Furthermore, CD1a is associated with relatively a favorable prognosis, and CD1a+ patients rarely relapsed or are refractory to treatment.

Others, such as CCR4 (C–C chemokine receptor type 4) and TRBC (T-cell receptor beta constant), are also expressed in some T-ALL and can be potential targets for T-ALL immunotherapy (94, 95).

### Antigen targets of CAR-T therapy in AML

#### CD123

One potential target for AML is CD123, an IL-3 receptor alpha chain that acts as a high-affinity receptor for stem cell factor (SCF) and is expressed at low levels in early hematopoietic cells, such as hematopoietic stem/progenitor cells (HSPCs) (96). CD123 is expressed in approximately 97% of AML patients and is overexpressed in 45% of patients, and at low levels by HSPCs, monocytes, a subset of dendritic cells, and endothelial cells (97–99). As early as 2013, Tettamanti et al. designed CD123 CAR-infected cytokine-induced killer cells (CIKs). These CIKs showed efficacy against CD123+ THP1 cell line and primary AML blasts, with minimal effects on healthy monocytes and endothelial cells with low expression of CD123, which proves the feasibility of CD123 as a therapeutic target (100). In 2014, Mardiros et al. demonstrated that CD123 CAR-T cells were effective in eliminating CD123+ LCL, KG-1a cell lines, and primary AML blasts without eliminating granulocyte/macrophage and erythroid colony formation *in vitro*. Notably, patients-derived CAR-T cells could eliminate autologous AML blasts *in vitro (* 101). Gill et al. found that CD123 CAR was efficient in clearing human primary leukemia cells, meanwhile inducing myeloablation in xenograft mouse models, which suggests that CD123 CAR-based myeloablation may be used to bridge HSCT (102). Due to CD123 and CD33, both are expressed on AML cells and normal HSPCs, Pizzitola et al. modified CKI cells with the CD123 and CD33 CAR to compare their efficacy and safety. Both these CAR-T cells efficiently eliminated primary human AML KG-1a cells in mice, but only CD123 CAR showed limited killing efficacy on normal HSPCs compared to CD33 CAR (103). In 2015, Luio et al. first treated one relapsed AML-M2 (FLT3/ITD+), male patient with apoptosis-inducible CD123 CAR, and the patient subsequently achieved partial remission within 20 days. Although CRS occurred on day 4, it was effectively controlled with a single dose of Tocilizumab (104). To address myeloablation after CD123 CAR-T therapy in AML, Tasian et al. compared three CAR-T-cell clearance strategies: (1) transiently active anti-CD123 messenger RNA electroporated CAR T cells (RNA-CART123); (2) T-cell clearance with alemtuzumab after CD123 CAR-T-cell therapy; and (3) T cell ablation with rituximab to CD20-coexpressing CART123(CART123-CD20) after therapy. The author found that CD123 CAR-T cells showed strong antitumor activity to MOLM14 cell line and primary AML blasts in mouse xenograft models, and all these three approaches could efficiently deplete CAR-T cells without affecting antileukemic effects. Notably, the ablation of CAR-T cells allowed subsequent HSCT rescue in normal hematopoiesis xenograft models (105). Thus, strategies for posttreatment CAR-T cell clearance may be effective to alleviate detrimental side effects, such as myeloablation. Further to this area of investigation, Arcangeli et al. designed a rational mutation in the anti-CD123 CAR antigen binding domain to reduce the binding affinity of CAR, which could minimize the toxicity and side effects against normal tissues with low expression of CD123 without affecting the antileukemic activity to tumor cells; altogether demonstrating the manageable safety of CD123 CAR in AML (106). The same year (2017), Budde et al. reported in an abstract form that a Phase I dose escalation clinical trial, which evaluated the efficacy and safety of CD123 CAR in 6 patients with refractory AML after treatment with autologous HSCT (alloHSCT). In this study, 4 of 6 patients achieved CR, and 2 patients reported reduced blast counts but did not achieve remission after a single or second infusion of CAR-T cells. Notably, all side effects were reversible and controllable (107). Meanwhile, to ablate CAR-T cells after therapy in patients, Cummins et al. evaluated the safety of CD123 CAR in R/R AML patients with mRNA electroporate technology. CART123 cells did not expand successfully *in vivo*, and all 5 patients treated with mRNA CART123 eventually developed clinical progression (108). To improve the efficacy and persistence of CAR-T cells *in vivo*, in 2018, Mu et al. developed CD123 CAR-expressing IL-15. The results showed that genetically engineered CD123 CAR improved the anti-AML activity against CD123+ cell lines *in vitro* and eliminated primary AML cells *in vivo (* 109). Loff et al. redirected CD123 CAR-T cells using a switch-controllable universal CAR T platform (UniCAR) based on two major elements: a non-reactive inducible CAR and a soluble targeting module (TM) enabling UniCAR-T reactivity in an antigen-specific manner. UniCAR T 123 exhibited potent cytotoxic activity against patient derived CD123+ leukemia cells *in vitro* and *in vivo*. Notably, in this study, the activation, cytolytic activity, and cytokine release profiles for UniCAR T123 were all tightly controlled. Compared with traditional CD123 CAR-T cells, UNICAR T 123 cells can additionally distinguish malignant leukemia cells with high CD123 expression from healthy tissues with low CD123 expression, features that further improve the safety of CD123 CAR (110). A phase I clinical trial of UNICAR-CD123-CAR is currently in progress (111). In 2019, Qin et al. developed a simple and highly selective D-domain, which was derived from *de novo*-designed α-helical bundle-α3D, to target CD123 for a unique CD123 CAR. The work revealed that CD123 CAR composed of D-domain mediated efficiently mediated T-cell activation and cytotoxicity to MOLM14, IM9, KG-1a, and NALM6 cell lines, and induced complete and durable remission in two AML xenograft mouse models. This work supports the development of multifunctional CARs through such an approach (112). Yao et al. used donor-derived CD123 CAR T cells as a conditioning regimen for haploidentical HSCT (haploid-HSCT) in a patient with FUS-ERG+ AML and found that CD123 CAR-T cells reduced chemotherapy-resistance blasts without affecting donor chimerism and myeloid implantation (113). To enhance the anti-tumor function of CD123 CAR-T cells, in 2020, You et al. combined decitabine treatment with CD123 CAR for AML. The results showed that decitabine enhances the anti-leukemia efficacy of CD123 CAR-T cells to THP1 cell line *in vitro* and *in vivo*, alongside CD123 CAR-T cells differentiate into naive and memory phenotypes (114). In 2021, UniCAR-T-123 with the targeting module TM123 was used to treat 3 R/R AML patients. 1 patient showed partial remission and 2 patients showed CR and adverse events were generally mild (115). This clinical trial is still ongoing. To minimize the side effect on normal hematopoietic progenitor cells and prolong the persistence of CD123 CAR-T cells, Khawanky et al. found that demethylating therapy could increase the CD123 expression on leukemia cells and increase CTLA4- CD123 CAR-T cell proportion and showed superior cytotoxicity against AML cells, accompanied by higher TNFα production in leukemia-bearing mice (116). Due to CD123 could distinguish HSC from leukemia stem cells(LSCs), to eliminate LSCs and preserve normal HSC, in 2022, TALEN gene-editing technology was used to produce a TCRαβ negative allogeneic CD123 CAR (UCART123), which preferentially eliminates primary AML than normal cells with modest toxicity *in vitro (* 117). While CAR targeting CD123 has shown efficacy and safety in preclinical and some clinical trials, but myeloablation occur after CAR-T therapy. Therefore, some strategies such as transiently active messenger RNA for CAR, alemtuzumab or rituximab treatment, and UniCAR may be utilized to ablate CAR-T cells *in vitro* and *in vivo*. The depletion of CAR-T cells after therapy requires further investigation in clinical studies. Nevertheless, the safety and efficacy profiles of CAR therapy targeting CD123 are reflected in the finding that 7 clinical trials are recruiting to explore its feasibility to treat AML, as shown in **Table 4**.

**Table 4:** Recruiting CD123 CAR clinical trials.

NCT Number	Interventions	First posted	Phase	Locations
NCT03190278	Allogeneic UCART123v1.2	16-Jun-17	I	United States
NCT04010877	CLL-1, CD33 and/or CD123-specific CAR gene-engineered T cells	8-Jul-19	I/II	China
NCT04265963	CD123 CAR-T cells	12-Feb-20	I/II	China
NCT04272125	CD123 CAR-T cells	17-Feb-20	I/II	China
NCT04230265	UniCAR02-T	18-Jan-20	I	Germany
NCT04318678	CD123-CAR T	24-Mar-20	I	United States
NCT04678336	CART123 cells	21-Dec-20	I	United States

#### CD33

CD33 is a transmembrane receptor of the sialic-acid-binding immunoglobulin-like lectin (SIGLEC) family and is expressed on myeloid cells ranging from progenitors to well-differentiated cells, including neutral granulocytes, monocytes, and tissue-resident macrophages (118). However, in pathological states, CD33 is expressed in approximately 80-90% of AML patients and may also be expressed on LSCs (119–122). In 2010, to improve the effector functions of CIK cells, Marin et al. transduced anti-CD33-ζ and anti-CD33-CD28-OX40-ζ CARs into CIK cells and found that the CD33 CAR enhanced the anti-leukemic functions of CIK cells against HL60 and KG-1a cell lines and primary AML blasts (123). In 2012, to decrease tumor escape, Dutour et al. transduced human Epstein Barr virus (EBV)-specific cytotoxic T cells with CD33 CAR, and the CAR-T cells displayed EBV and HLA-unrestricted bispecificity *in vitro* and anti-AML tumor activity in CD33+ human AML-bearing mice without irreversibly disrupting the formation of CD34(+) hematopoietic progenitor clones (124). In 2014, Pizzitola compared the cytotoxicity of CD123 and CD33 CAR on AML, and find that no difference in anti-leukemic activity, yet CD33 CAR appeared to have stronger cytotoxicity on normal HSPCs compared with CD123 CAR (103). In 2015, Wang et al. reported an autologous CD33 CAR in one R/R AML patient, with no uncontrollable clinical toxicities, but with subsequent disease progression at 9 weeks post-T-cells infusion (125). Kenderian and colleagues used the scFv of *gemtuzumab ozogamicin* (clone My96) to develop a CD33 CAR (CART33). CART33 exhibited significant cytotoxicity against the MOLM14 cell line *in vitro* and eradication of leukemia in AML xenograft, yet a reduction of myeloid progenitors in xenograft models was also observed. Thus, the author prepared transient CART33 cells by expressing modified mRNA, which exhibited potent but self-limited activity against AML (126). To minimize the myelosuppression after CAR-T therapy, in 2016, Minagawa et al. designed Caspase9-CAR CD33T cells inducibly selected by ΔCD19, which could specifically lyse CD33+ MV4-11 tumor cells and primary leukemic blasts *in vitro*, following which CAR-T cells were largely eliminated by suicide gene activation (127). In 2018, Kim et al. proved that CD33 KO HSPC showed normal engraftment and differentiation in the mouse model, and autologous rhesus macaques CD33 KO HSPC showing normal myeloid function *in vivo*. Most importantly, CD33 CAR-T cells showed efficient elimination of leukemia, while CD33-deficient cells were spared without myelotoxicity, as observed in human xenograft models (128). Li et al. demonstrated that 4-1BB as a costimulator could endow CAR-T cells with increased central memory and prolonged survival for maximum efficacy, as compared to CD28, and both CD28 and 4-1BB (129). Due to PI3K pathway being activated in CD33 CAR-T cells, to increase the persistence of CAR-T cells *in vivo*, Zhang et al. used PI3K inhibitors to modulate the differentiation of CD33 CAR-T cells. The data showed that it maintained CAR-T cells at a lower differentiated state without affecting CAR-T-cell expansion (130). Schneider et al. constructed a humanized CAR33VH CAR, comprised of a human heavy-chain variable fragment, that exhibited antitumor activity to CD33 high expressed MOLM14 and HL60 cell lines and eliminated tumors in a MOLT-14 AML mouse model (131). In 2021, Tambaro and colleagues conducted a Phase I clinical trial to evaluate the efficacy and safety of CD33 CAR-T in R/R AML patients. It was reported that three patients received one low dose of CAR-T cells, and biologic activity was observed by associated symptoms and increased cytokine levels, however, an anti-leukemic response was not documented, and no dose-limiting toxicities were observed (132). To explore the impact of costimulatory domains on the CD33 CAR-T cells, Qin et al. developed six CD33-targeted CARs with one of three scFv of clinically tested CD33 antibodies, paired with CD28 or 4-1BB costimulatory domains. These six CARs exhibited cytotoxicity against CD33+ AML cell lines *in vitro* and *in vivo* and showed strong anti-leukemia activity against patient-derived xenograft (PDX) derived from pediatric AML patients. Furthermore, CD28-based CD33 CAR-T cells exhibited superior anti-leukemia compared with 4-1BB, and the safety and efficacy in patients were evaluated in a phase I clinical trial (133). In 2022, Liu et al. proved that the third generation CD33 CAR-T showed stronger vitality, proliferation ability, and stronger cytotoxicity than the second generation CAR. Notably, the third generation CD33 CAR-T preferentially killed leukemia cells while sparing CD33-deficient HSPCs (134). CD33 CAR showed efficient anti-AML activity *in vitro*, different costimulators, different generation CAR structures, and PI3K inhibitors may affect the anti-tumor activity, proliferation, and persistence of CD33 CAR-T cells. The efficacy of CD33 CAR in patients did not show promising results in some previous studies, more efforts are needed to improve the anti-tumor response of CD33 CAR. 6 clinical trials are recruited to explore the efficacy and safety of CD33 CAR in AML patients, as shown in **Table 5**.

**Table 5:** Recruiting CD33 CAR clinical trials.

NCT Number	Interventions	First posted	Phase	Locations
NCT03795779	CLL1-CD33 CAR T cells	8-Jan-19	I	China
NCT04010877	CLL-1, CD33 and/or CD123 CAR T cells	8-Jul-19	I/II	China
NCT03971799	CD33 CAR T cells	3-Jan-19	I/II	United States
NCT04835519	Functionally enhanced CD33 CAR T cells	8-Apr-21	I/II	China
NCT05248685	Dual CD33/CLL1 CAR T cells	21-Feb-22	I	China
NCT03927261	PRGN-3006 T cells	25-Apr-19	I	United States

#### CLL-1

C-type lectin-like molecule-1 (CLL-1) is reportedly expressed in more than 80% of AML blasts, and LSCs. Its expression is restricted to the myeloid lineage and absent in normal CD34+CD38- HSCs. Significantly, CLL1 is present on a small subset of chemotherapy-resistant LSCs (135–137), suggesting that it may be a potential target for therapeutic intervention. In 2017, Tashiro et al. first designed a CLL1 CAR with specific killing efficacy on CLL1+ HL60 and THP1 cell lines and primary AML blasts *in vitro*, as well as exhibited anti-leukemia activity in an HL60-AML xenograft mouse model. Notably, CLL1 CAR-T cells eliminated mature normal myeloid cells yet selectively spared healthy HSCs, to allow immune recovery after therapy (138). Laborda and colleagues designed an scFv-based CLL1 CAR that showed cytotoxic activity against HL60 and MOLM14 AML cell lines and patient-derived AML blasts *in vitro*, as well as clearance against tumor cells in mouse xenografts, while without damaging healthy HSCs. Compared with CAR designs utilizing CD8 as a hinge domain, IgG4 induced higher cytotoxicity in cell lines (139). In 2018, Wang et al. constructed a new CLL1 CAR using scFv from C57BL/6 mouse-derived CLL antibody, which has good anti-leukemia activity to U937 cell line and primary AML blasts *in vitro* and eliminated human AML in xenograft mouse models without targeting normal HSCs (140). Liu et al. first explored the safety and efficacy of CLL1 CAR in patient. A compound CAR targeting CLL1 and CD33 was constructed and allennimumab was used to clear CAR-T cells after tumor eradication. A 6-year-old patient with a complex karyotype of FLT3-ITD mutation received two split doses of CAR-T cells and achieved CR on day 19, followed by HSCT (141). In 2019, Atilla and colleagues explored the impact of different combinations of spacers, transmembrane, and intracellular signaling domains to CLL-1 CAR. By comparing their proliferation, functional persistence, and antitumor activity *in vitro* and *in vivo*, the data showed that CD28z CAR with a short hinge region or with a CD8 intracellular domain is better than others for CLL1 CAR (142). In 2020, Zhang et al. reported the case of one 10-year-old patient with secondary AML treated with CLL CAR-T cells. This patient finally achieved morphological, immunophenotypic, and molecular CR over 10 months, which provides a new treatment option for secondary AML (143). Atilla et al. demonstrated that CLL-1 CAR T cells with additional transgenic IL15 supplementation, and combined with a TNFα blocker antibody as well as activation of caspase-9 control switcher increased expansion, persistence, and anti-leukemia of CLL1 CAR-T cells in PDX and HL60 xenograft mouse models while avoided excessive cytokine production (144). PD-1 expression was increased after the activation of CAR-T cells and caused T cells exhaustion, to evaluate the efficacy of combination of PD-1 silencing and CLL-1 CAR-T therapy, in 2021, Lin et al. designed a PD-1-silenced CLL-1 CAR and reported that PD-1 silencing enhanced the cytotoxicity of CLL-1 CAR (145). Similar to this, Zhang et al. designed a CLL-1 CAR based on the apoptosis-inducing gene FKBP-Caspase9 and evaluated the efficacy and safety in four R/R AML patients. The author reported that three patients achieved CR with MRD negativity, while the fourth survived for 5 months, with manageable side effects (146). CLL1 CAR showed promising anti-tumor efficiency in pre-clinical experiments and anti-AML response in AML patients and selectively spared normal HSCs. There are 8 clinical trials for CLL-1 CAR currently recruiting, as shown in **Table 6**.

**Table 6:** Recruiting CLL1 CAR clinical trials.

NCT Number	Interventions	First posted	Phase	Locations
NCT04884984	anti-CLL1 CAR T cells	13-May-21	I/II	China
NCT03795779	CLL1-CD33 cCAR T cells	8-Jan-19	I	China
NCT04010877	CLL-1, CD33 and/or CD123 CAR T cells	8-Jul-19	I/II	China
NCT04219163	CLL-1 CAR T cells	6-Jan-20	I	United States
NCT04789408	KITE-222	9-Mar-21	I	United States
NCT04923919	Anti-CLL1 CAR T cells	1-Jun-21	I	China
NCT05248685	Dual CD33/CLL1 CAR T cells	21-Feb-22	I	China
NCT05252572	CLL1 CAR T cells	23-Feb-22	I	China

#### CD70

CD70 is a tumor necrosis factor (TNF) receptor ligand and is proven to be absent in normal tissues and hematopoietic cells, but highly expressed on most AML blasts and AML stem/progenitor cells (147, 148). In 2017, Riether et al. reported that CD70/CD27 signaling promotes blast stemness, and blocking CD70/CD27 by mAb could prolong survival in murine AML xenografts, representing that CD70/CD27 is a promising therapeutic strategy for AML (149). CD19 CAR-T cell therapy has presented revolutionary progression in CD19+ hematological malignancies, but some patients reccurence due to the exiting of CD19- tumor cells (150). Therefore, it is necessary to develop alternative antigens to avoid antigen escape. In 2021, Deng et al. designed L/H and H/L svFv-based CD70 CAR, truncated CD27-based CD70 CAR and anti-CD19 CAR as controls. The results showed that anti-CD70 (H/L) effectively killed CD19+ and CD19- Raji cells *in vitro* and in NSG xenograft mouse models, altogether providing a new therapeutic option for patients who have CD19- recurrence (151). Sauer et al. compared a panel of scFv-based CD70 CARs with the same scFv and different size and flexibility of the extracellular spacer, different transmembrane, and different costimulatory domains to CD27-based CD70 CAR. The results showed that the ligand CD27-based CD70 CAR presents superior proliferation and antitumor activity against AML cell lines Molm-13, THP-1, and IMS-M2 *in vitro* and Molm-13 AML mouse xenografts and primary AML *in vivo* (152). In 2022, Leick et al. used azacitidine to increase antigen density of CD7 in tumor cells and designed a CD8 hinger and transmembrane-modified CD27-based CD70 CAR to mitigate cleavage of the extracellular portion of CD27, altogether could enhance avidity and expansion of CD70 CAR-T cells and lead to more potent activity *in vivo* (153). The safety and efficacy of CD70 CAR in patients need more exploration in the clinical trial. There are only one clinical trial about CD70 CAR-T in AML currently recruiting as shown in **Table 7**.

**Table 7:** Recruiting CD70 CAR clinical trials.

NCT Number	Interventions	First posted	Phase	Locations
NCT004662294	CD70 CAR T-cells	10-Dec-20	I	China

In addition, some antigens such as CD38, FLT3 et al., can be potential targets for AML CAR-T therapy. The recruiting clinical trials are shown in **Table 8**.

**Table 8:** Recruiting other CAR clinical trials for AML.

NCT Number	Interventions	First posted	Phase	Locations
NCT05023707	Anti-FLT3 CAR-T	26-Aug-21	I/II	China
NCT05432401	FLT3 CAR-T	27-Jun-22	I	China
NCT05017883	FLT3 CAR-T	24-Aug-21	NA	China
NCT05488132	Anti-siglec-6 CAR-T	4-Aug-22	I/II	China
NCT04692948	CD276 CAR-T	5-Jan-21	NA	China
NCT04169022	IL1RAP CAR-T	19-Nov-19	NA	France
NCT04351022	CD38 CAR-T	17-Apr-20	I/II	China
NCT04662294	CD70 CAR T	10-Dec-20	I	China
NCT04803929	Anti-ILT3 CAR-T	18-Mar-21	I	China

## Summary

Compared with B-ALL, T-ALL and AML are forms of leukemia that display more complex morphological features and are associated with poor prognosis. Furthermore, T-ALL and AML are associated with fewer treatment options after relapse or refractory. Given the significant success of CAR-T-cell therapy in B-cell malignancies, a similar approach for non-B-cell acute leukemia appears to represent a promising direction for the development of improved treatments. Although CAR-T therapy for non-B-cell leukemia still faces great challenges, researchers are already exploring multiple therapeutic targets, with promising results in preclinical and clinical studies, such as with CD7, CD5, CD4, and other targets in T-ALL that overcome CAR-T-cell fratricide, tumor cell contamination and T-cell immunodeficiency. Furthermore, targets such as CD123, CD33, CLL1, and CD70 also show great promise for the treatment of AML. Taken altogether, the collective efforts of researchers and clinicians to develop CARs and deliver them in current clinical trials will fulfill the promise to find effective treatments for non-B-cell leukemia.

## Author contributions

WW drafted the original manuscript and designed the figures. DY, XC and DL edited the manuscript. LZ and XZ reviewed, revised, and supervised the work.

## Funding

This work was financially supported by the National Natural Science Foundation of China (No. 82172701).

## Acknowledgments

Manuscript editor Julian Heng (Remotely Consulting, Perth, Australia) provided professional English-language editing of this article.

## Conflict of interest

The authors declare that the research was conducted in the absence of any commercial or financial relationships that could be construed as a potential conflict of interest.

## Publisher’s note

All claims expressed in this article are solely those of the authors and do not necessarily represent those of their affiliated organizations, or those of the publisher, the editors and the reviewers. Any product that may be evaluated in this article, or claim that may be made by its manufacturer, is not guaranteed or endorsed by the publisher.
